# Effect of LncRNA-MALAT1 on mineralization of dental pulp cells in a high-glucose microenvironment

**DOI:** 10.3389/fcell.2022.921364

**Published:** 2022-08-11

**Authors:** Xinzhu Li, Wenan Xu, Xiaoyu Lin, Jingyi Wu, Buling Wu

**Affiliations:** ^1^ Department of Stomatology, Nanfang Hospital, Southern Medical University, Guangzhou, China; ^2^ Department of Pediatric Dentistry, Shenzhen Stomatology Hospital (Pingshan), Southern Medical University, Shenzhen, China; ^3^ Stomatological Hospital, Southern Medical University, Guangzhou, China

**Keywords:** LncRNA-long noncoding RNA, lncRNA-MALAT1, dental pulp, high glucose, mineralization

## Abstract

Metastasis-associated lung adenocarcinoma transcript 1 (MALAT1) belongs to the long non-coding RNA (LncRNA) family. LncRNA-MALAT1 is expressed in a variety of tissues and is involved in a variety of diseases and biological processes. Although LncRNA-MALAT1 is upregulated in a high-glucose microenvironment and may participate in odontogenic differentiation, the underlying mechanism is not yet well elucidated. Here, we show that MALAT1 was mainly expressed in the cytoplasm of dental pulp cells (DPCs) *in situ* hybridization. In addition, high levels of mineralization-related factors, namely, tumor growth factors *β* 1 and 2 (TGFβ-1 and TGFβ-2), bone morphogenetic proteins 2 and 4 (BMP2 and BMP4), bone morphogenetic protein receptor 1 (BMPR1), SMAD family member 2 (SMAD2), runt-related transcription factor 2 (RUNX2), Msh homeobox 2 (MSX2), transcription factor SP7 (SP7), alkaline phosphatase (ALP), dentin matrix acidic phosphoprotein 1 (DMP1), and dentin sialophosphoprotein (DSPP), were expressed, and MALAT1 was significantly overexpressed in DPCs 7 and 14 days after mineralization induction in a high-glucose microenvironment, but only TGFβ-1, BMP2, MSX2, SP7, ALP, and DSPP were significantly downregulated in DPCs after MALAT1 inhibition. MALAT1 may participate in the mineralization process of DPCs by regulating multiple factors (TGFβ-1, BMP2, MSX2, SP7, ALP, and DSPP).

## Introduction

Diabetes (types 1 and 2) is a metabolic disease characterized by a chronic increase in blood glucose levels. Common complications of diabetes are retinopathy, kidney disease, neuropathy, microvascular disease, infection, and impaired wound healing. High blood glucose levels are the most typical manifestation of diabetes. Poor glycemic control can lead to the formation of advanced glycation end products (AGEs) and glycated hemoglobin (Vlassara, 1997). AGEs are products of non-enzymatic reactions between glucose and free amino acid residues in proteins. These products lead to the thickening of the capillary basement membrane. Diabetes can damage blood vessels, especially capillaries, mostly *via* atherosclerotic deposition in the intimal tissue of the vascular lumen. Damaged capillaries then form a thickened basement membrane, which can initiate interleukin response (Witko-Sarsat and Deschamps-Latscha, 1994). The pulp is located in the pulp cavity of the tooth, which mainly includes the nerves, blood vessels, lymph, and connective tissue. Capillaries are the main vessels in pulp; thus, when they are exposed to a high-glucose environment for long periods, they become damaged and develop lesions. The pulp is prone to irreversible inflammation because it has a vascular system but no collateral vessels; it is located in the dentin and cannot change in volume. One study reported that the pulps of rats were exposed by 1/16th surgical round burs and covered with the MTA, and self-healing ability was reduced in diabetic rats compared with non-diabetic rats (Garber et al., 2009). Therefore, it is very important to study how pulp tissue promotes dentin restoration in a high-glucose environment. The expression of vascular endothelial growth factor (VEGF) and bone morphogenetic protein 2 (BMP2) is significantly higher in patients with diabetes (Ilić et al., 2012). Rats with induced diabetes develop systemic myeloid necrosis and severe periapical lesions after 6 months (Claudino et al., 2015). These findings indicate that diabetes is associated with both periodontal and pulp diseases.

Some LncRNAs not only encode proteins but also participate in vital activities of organisms *via* a complex regulatory mechanism. These RNAs can combine with DNA and proteins and form complexes with other RNAs involved in transcriptional and post-transcriptional regulation (Mercer et al., 2009; Ghosal et al., 2013). Diabetes, as a major metabolic disease, results in a variety of complications affecting most major organs. It has been established that LncRNAs participate in the development of diabetic complications, such as diabetic nephropathy (Guo et al., 2019), diabetic retinopathy (Shan et al., 2017), diabetic neuropathic pain (Liu et al., 2017), and so on. Metastasis-associated lung adenocarcinoma transcript 1 (MALAT1) is an important LncRNA, as it is activated under anoxia, hyperglycemia (Liu et al., 2014; Shaker et al., 2019; Cheng et al., 2020; Han et al., 2020), ultraviolet radiation, infection, and chemical stimulation and is involved in regulating cell proliferation (Zhang et al., 2018), apoptosis (Gu et al., 2017), differentiation, migration, epithelial–mesenchymal transformation (Lee et al., 2017), autophagy, and maintaining cellular morphology. Evidence suggests that LncRNA-MALAT1 plays a key role in diabetic complications. The knockdown of MALAT1 reduced human retina microvascular endothelial cell proliferation, migration, tube formation, and vascular permeability by upregulating miR-125 b in a high-glucose microenvironment (Liu et al., 2019). In a high-glucose microenvironment, MALAT1 reduces retinitis and regulates endothelial cell function *via* the p38MAPK signaling pathway (Liu et al., 2014). A study has shown that MALAT1 may promote odontogenic differentiation of dental pulp stem cells (DPSCs). MALAT1 can induce odontoblast differentiation of DPSCs by downregulating the expression of miR-140-5p, resulting in the high expression of GIT215 (Bao et al., 2020). This is only a small part of how MALAT1 participates in the process of odontogenic differentiation. Its regulatory network is diversified, so it is very important to understand its regulatory mechanism. Our research will be a supplement to its regulatory mechanism.

Although LncRNA-MALAT1 is upregulated in the high-glucose microenvironment and may participate in odontogenic differentiation, the underlying mechanism is not yet well elucidated. Because pulp tissues of patients with diabetes are prone to mineralization, the present study aimed to determine whether LncRNA-MALAT1 is involved in regulating DPC mineralization in a high-glucose environment.

## Materials and methods

### Cell culture

DPCs were isolated and cultured using the method described in a previous study (Li et al., 2014). Dental pulp was separated from healthy premolars or third molars which are extracted from patients 12–25-years old for orthodontics or impaction. The DPCs were passaged until they were 80% confluent. Cell phenotype analysis was performed by flow cytometric analysis for CD90/PE, CD29/PE, and CD44/FITC (PharMingen-BD Biosciences, San Diego, CA) (Supplementary Figure S1).

### Effects of a high-glucose microenvironment on dental pulp cell proliferation and cloning

DPCs were inoculated on 96-well plates at a density of 1 × 10^3^ cells and divided into a high-glucose group (DMEM containing 10% FBS, 50 mmol/L glucose) and an NC group (DMEM containing 10% FBS). The proliferation ability of the DPCs was detected by MTT assay.

The DPCs were inoculated in a 60-mm culture dish at a density of 1 × 10^3^ cells and divided into two groups (high-glucose group and NC group). After 14 days, the clonal forming ability of DPCs was detected by crystal violet staining after fixation with 4% paraformaldehyde at room temperature for 30 min.

### Quantitative real-time polymerase chain reaction

Total isolated messenger RNA (mRNA) was served as the template to generate complementary DNA through reverse transcription using a reagent kit (Vazyme, CHN). The primer sequences of mineralization-related factors are shown in Table 1. The quantitative real-time polymerase chain reactions (qRT-PCRs) were performed using the SYBR^®^ Premix Ex Taq™ II (TaKaRa) on an ABI Q5 real-time polymerase chain reaction system (ABI, Carlsbad, CA). Relative quantization was performed by determining the difference between the threshold cycle (Ct) of GAPDH and the Ct of each transcript and computing DDCt. Amplification proceeded as per the manufacturer’s instructions.

**TABLE 1:** Primer sequences used for qRT-PCR.

Primer	Sequences
TGF-β1	5′-CAA​TTC​CTG​GCG​ATA​CCT​CAG-3′
	5′-GCA​CAA​CTC​CGG​TGA​CAT​CAA-3′
TGF-β2	5′-CCA​TCC​CGC​CCA​CTT​TCT​AC-3′
	5′-AGC​TCA​ATC​CGT​TGT​TCA​GGC-3′
BMP2	5′-TGG​ACG​CTC​TTT​CAA​TGG​AC-3′
	5′-AGC​AGC​AAC​GCT​AGA​AGA​C-3′
BMP4	5′-GAG​CCT​TTC​CAG​CAA​GTT​TG-3′
	5′-CCC​GTC​TCA​GGT​ATC​AAA​CTA​G-3′
BMPR1	5′-AAA​ACC​ACT​TCC​AGC​CCT​AC-3′
	5′-ACA​CAA​CCT​CAC​GCA​TAT​CTT​C-3′
SMAD2	5′-GAT​CCT​AAC​AGA​ACT​TCC​GCC-3′
	5′-CAC​TTG​TTT​CTC​CAT​CTT​CAC​TG-3′
RUNX2	5′-TGG​TTA​CTG​TCA​TGG​CGG​GTA-3′
	5′-TCT​CAG​ATC​GTT​GAA​CCT​TGC​TA-3′
MSX2	5′-CGG​TCA​AGT​CGG​AAA​ATT​CAG-3′
	5′-GGA​TGT​GGT​AAA​GGG​CGT​G-3′
SP7	5′-CCT​CTG​CGG​GAC​TCA​ACA​AC-3′
	5′-AGC​CCA​TTA​GTG​CTT​GTA​AAG​G-3′
ALP	5′-CCA​GGG​CTG​TAA​GGA​CAT​C-3′
	5′-GGC​TTT​CTC​GTC​ACT​CTC​ATA​C-3′
DMP1	5′-CTC​CGA​GTT​GGA​CGA​TGA​GG-3′
	5′-TCA​TGC​CTG​CAC​TGT​TCA​TTC-3′
DSPP	5′-TTT​GGG​CAG​TAG​CAT​GGG​C-3′
	5′-CCA​TCT​TGG​GTA​TTC​TCT​TGC​CT-3′
MALAT1	5′-CTT​AAG​CGC​AGC​GCC​ATT​TT-3′
	5′-CCT​CCA​AAC​CCC​AAG​ACC​AA-3′
U6	5′-CTC​GCT​TCG​GCA​GCA​CA-3′
	5′-AAC​GCT​TCA​CGA​ATT​TGC​GT-3′
GAPDH	5′-GAC​AGT​CAG​CCG​CAT​CTT​CT-3′
	5′-AAA​TGA​GCC​CCA​GCC​TTC​TC-3′

### Western blot

The total protein was extracted from the cultured cells and separated by sodium dodecyl sulfate-polyacrylamide gel electrophoresis (SDS PAGE) and transferred onto a PVDF membrane (Bio-rad, United States). The membrane was incubated with rabbit monoclonal anti-BMP2 (1:1000; Proteintech, United States), rabbit monoclonal anti-BMP4 (1:1000; Affinity Biosciences, United States), rabbit monoclonal anti-DMP1 (1:1000; Abcam, United Kingdom), rabbit monoclonal anti-ALP (1:1000; Abcam, United Kingdom), mouse monoclonal anti-RUNX2 (1:1000; Abcam, United Kingdom), rabbit monoclonal anti-TGFβ (1:1000; Abcam, United Kingdom), rabbit monoclonal anti-DSPP (1:1000; Affinity Biosciences, United States), rabbit monoclonal anti-MSX2 (1:1000; Bioss, CHN), and rabbit monoclonal anti-GAPDH (1:1000; Abcam, United Kingdom) overnight at 4°C. Proteins were visualized using HRP-conjugated donkey anti-rabbit IgG (1:20000; BBI Life Sciences) and HRP-conjugated donkey anti-mouse IgG (1:20000; BBI Life Sciences) secondary antibody. The membrane was scanned on an Odyssey V3.0 scanner (Li-cor).

### Alizarin red stain

The DPCs were divided into two groups (high-glucose group and NC group), which were mineralized 7 and 14 days after induction. After washing with PBS three times, it was fixed with 4% paraformaldehyde at room temperature for 30 min and then using 2% alizarin red staining for 30 min. An appropriate amount of cetylpyridine chloride was added to each well, extracted to the 96-well plate after 1 h, and detected its OD value at the wavelength of 562. It is the semi-quantitative analysis after the mineralization induction of DPCs in two groups.

### *In Situ* hybridization assay

DPCs are made into cellular slivers which were cultured in high-glucose and mineralization induction on day 14 . The slides are placed in PBS three times, 5 min each time, then 0.3% Triton X-100 is applied in PBS, incubated at room temperature for 20 min, and washed in PBS. Sufficient 4% paraformaldehyde is dropped drop-wise to each specimen, incubated for 20 min at room temperature, and then pre-hybridization is begun. The probe with hybridization buffer (probe: hybridization buffer = 1 μl:50–200 μl) is diluted, denatured at 85 ± 2°C for 5 min, and the probes are kept at 37°C for 2 min. The pre-hybridization buffer is wiped off and the denatured probe is applied to the slide. A coverslip is applied immediately and sealed with rubber cement. The slide is kept in a moist chamber at 37–42°C overnight for hybridization. The coverslips are removed by submerging the slides in 2× SSC/0.1% Tween 20 and soaking the slide until the coverslips fall off. The slides are washed with PBS for 5 min. Sufficient 3% BSA solution is added drop-wise to each specimen and incubated for 60 min at 37°C. Dilute the Anti-Digoxin, Rhodamine Conjugated with 3% BSA. To each specimen, 2–3 drops of second antibody solution is added, and each specimen is covered with a coverslip and incubated for 60 min at 37°C (in the dark). The coverslips are removed by submerging the slides in PBS buffer and soaking the slide until the coverslips fall off. An amount of 20 μl DAPI-Antifade Solution is added, the slide is covered with a coverslip and incubated at room temperature for 15–20 min (in the dark). The slides are examined under a fluorescence microscope with a proper filter.

### Statistical analysis

The mineralization-related factors’ expression differences between different groups of cells were analyzed using two-sample *t*-test. The RQ values (2^−△△CT^) of the same factor in the induction group were compared with those of the NC group (RQ value was 1) by using two-sample *t*-test. Differences were considered significant at *p* < 0.05. All statistical analyses were conducted using SPSS 19.0 software (SPSS Inc., Chicago, IL).

## Results

### Effects of a high-glucose microenvironment on dental pulp cell proliferation and cloning

**FIGURE 1:**
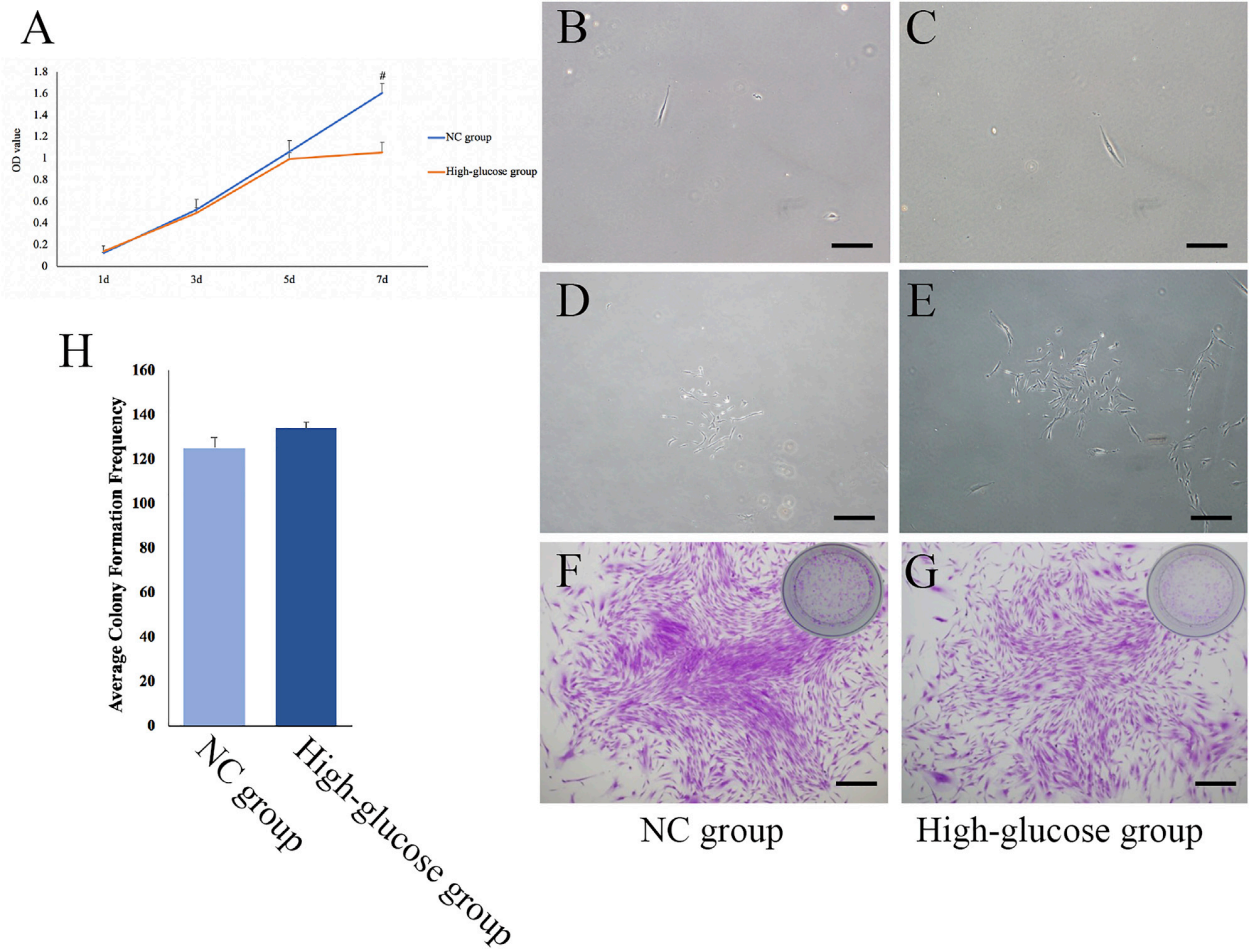
Effects of a high-glucose microenvironment on DPC proliferation and cloning. **(A)** Analysis of DPC proliferation ability in a high-glucose environment by MTT. **(B,C)** On the second day of inoculation, the DPCs were single-cell adherent. **(D,E)** After 7 days of inoculation, DPCs grew in a clonal manner. **(F,G)** After inoculation for 14 days, the cloned structure was stained with crystal violet, and the right corner was marked as the plate photo. **(H)** Histogram after quantitative determination (*n* = 3, scale bar = 500 μm ^*#*^ *p < 0.05*).

The results of the MTT assays showed that DPCs entered the logarithmic growth phase within 3 days, the high-glucose (HG) group entered the plateau phase after 5 days, and the normal controls (NC) did not obviously enter a plateau phase during the study. On day 7 of proliferation, the optical density (OD) of the NC and HG groups significantly differed (1.609,325 ± 0.13 and 1.054625 ± 0.1, respectively, *p < 0.05*; Figure 1A). The HG microenvironment inhibited DPC proliferation.

Microscopy analysis showed that the NC and HG groups of DPCs formed clones. Single DPCs on day 2 adhered to the wells (Figures 1B,C), following which both groups started to form clones on day 7 (Figures 1D,E). Crystal violet staining on day 14 revealed the structure of the clones (Figures 1F,G). The numbers of clones formed by the two groups of DPCs did not significantly differ (*p > 0.05*; Figure 1H). These results showed that DPC clone formation could be maintained in a high-glucose microenvironment.

### Effects of a high-glucose microenvironment on dental pulp cell mineralization

**FIGURE 2:**
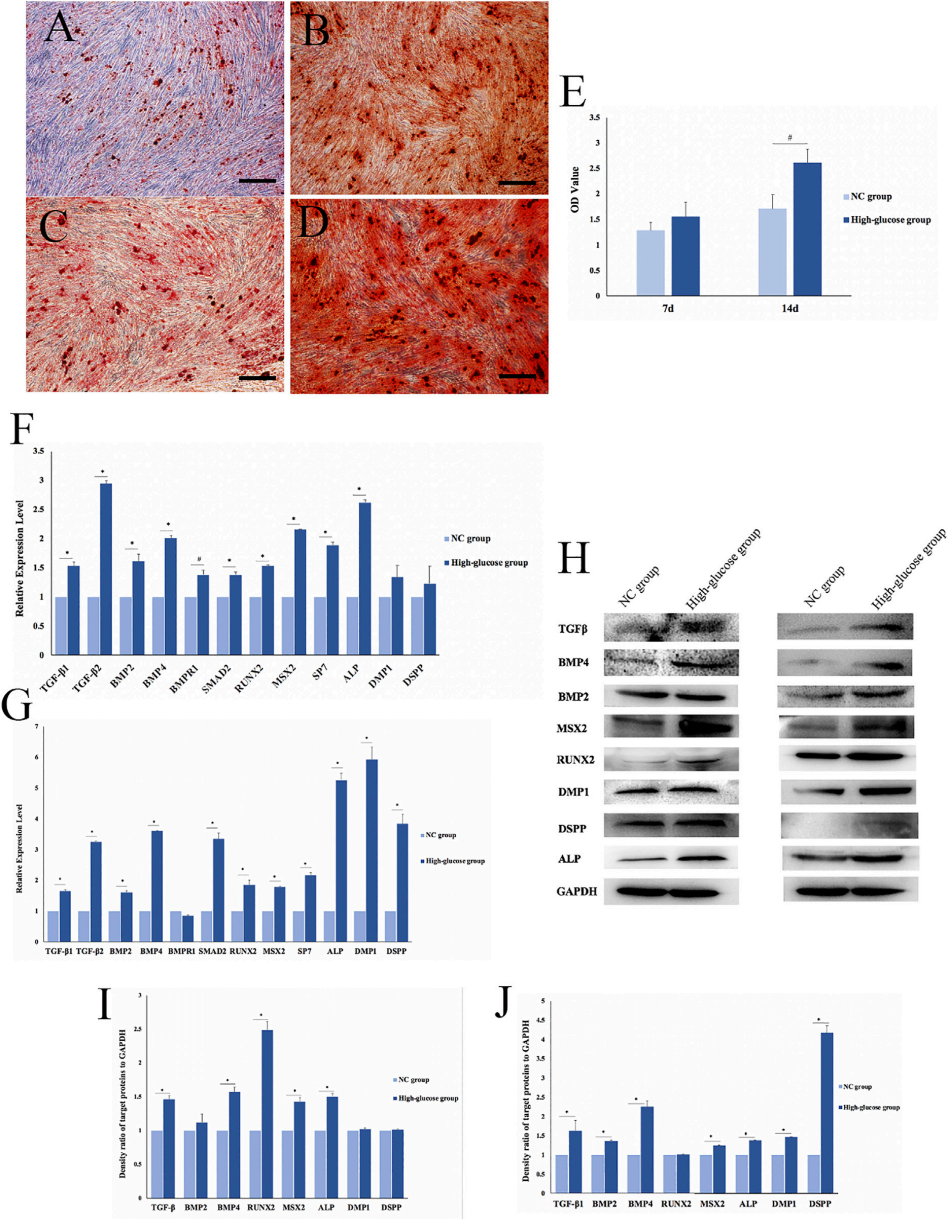
Effects of a high-glucose microenvironment on mineralization of DPCs. **(A,C)** NC group 7 /14 days after induced mineralization stained by alizarin red; **(B,D)** high-glucose group 7 /14 days after induced mineralization stained by alizarin red; **(E)** semi-quantitative analysis of alizarin red staining; **(F)** expression of mineralization-related factors was detected by qRT-PCR after 7 days of DPCs which were cultured in a high-glucose microenvironment; **(G)** expression of mineralization-related factors was detected by qRT-PCR after 14 days; **(H)** expression results of mineralization-related factors detected by Western blot after 7 /14 days of DPC induction in a high-glucose microenvironment; **(I,J)** density ratio of target proteins to GAPDH on 7 days and 14 days (*n* = 3, scale bar = 500 μm ^*#*^ *p < 0.05,* *p < 0.01).

Alizarin red staining showed that 7 days after DPC mineralization induction, both groups of DPCs began to form mineralized nodules (Figures 2A,C). After 14 days of mineralization, intensified staining revealed more mineralized nodules formed by DPCs in the HG group than in the NC group (Figures 2B,D). Semi-quantitative results showed that the OD values at 562 nm were 1.28 ± 0.16 (NC) and 1.55 ± 0.29 (HG) after 7 days, and 1.7 ± 0.29 (NC) and 2.6 ± 0.28 (HG) after 14 days. The OD values were significantly higher at both time points for HG than those for the NC group (*p < 0.05*; Figure 2E).

qRT-PCR results on day 7 of mineralization induction showed significantly higher concentrations of the mineralization-related factors, TGFβ-1, TGFβ-2, BMP2, BMP4, bone morphogenetic protein 2 (BMPR1), SMAD family member 2 (SMAD2), runt-related transcription factor 2 (RUNX2), Msh homeobox 2 (MSX2), SP7, alkaline phosphatase (ALP), dentin matrix acidic phosphoprotein 1 (DMP1), and dentin sialophosphoprotein (DSPP) in the HG group than in the NC group (^*#*^ *p < 0.05,* *p < 0.01; Figure 2F). Similarly, the expression of mineralization-related factors significantly increased after 14 days of mineralization induction, except for BMPR1 (^*#*^ *p < 0.05,* *p < 0.01; Figure 2G). The qRT-PCR results showed that DPCs expressed significantly higher levels of mineralization-related factors in a high-glucose microenvironment. Therefore, HG significantly promoted DPC mineralization.

We used Western blotting to determine the expression of mineralization-related factors expressed at the protein level, namely, TGFβ signaling pathway proteins (TGF-β, BMP2, and BMP4), transcription factors (RUNX2 and MSX2), and mineralization-related downstream regulators (ALP, DMP1, and DSPP). The results showed that the HG microenvironment significantly promoted the expression of mineralization-related factors in DPCs on days 7 and 14 after mineralization (*p < 0.01; Figure 2H). The findings quantified using ImageJ software were consistent with those obtained *via* qRT-PCR (Figures 2I,J).

We verified the effects of the HG microenvironment on DPC mineralization at RNA, protein, and functional levels. The expression of mineralization-related factors increased and more mineralized nodules were formed in the HG group than in the NC group.

### Effects of LncRNA-MALAT1 on dental pulp cell mineralization

**FIGURE 3:**
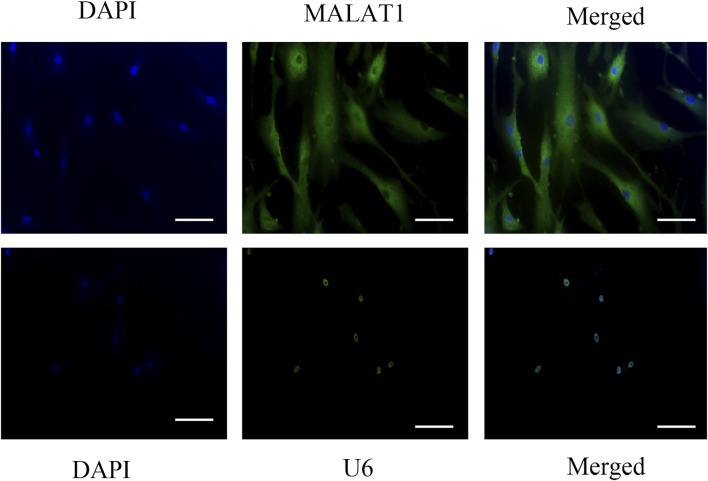
Insitu hybridization showed that LncRNA-MALAT1 was mainly expressed in the cytoplasm of DPCs (scale bar = 100 μm).

The *in situ* hybridization assay might help us understand the localization of gene expression, so we use it to explore where MALAT1 is expressed in DPCs. *In situ* hybridization findings showed high levels of MALAT1 expression in the cytoplasm of DPCs cultured for 14 days after hyperglycemia mineralization (Figure 3).

**FIGURE 4:**
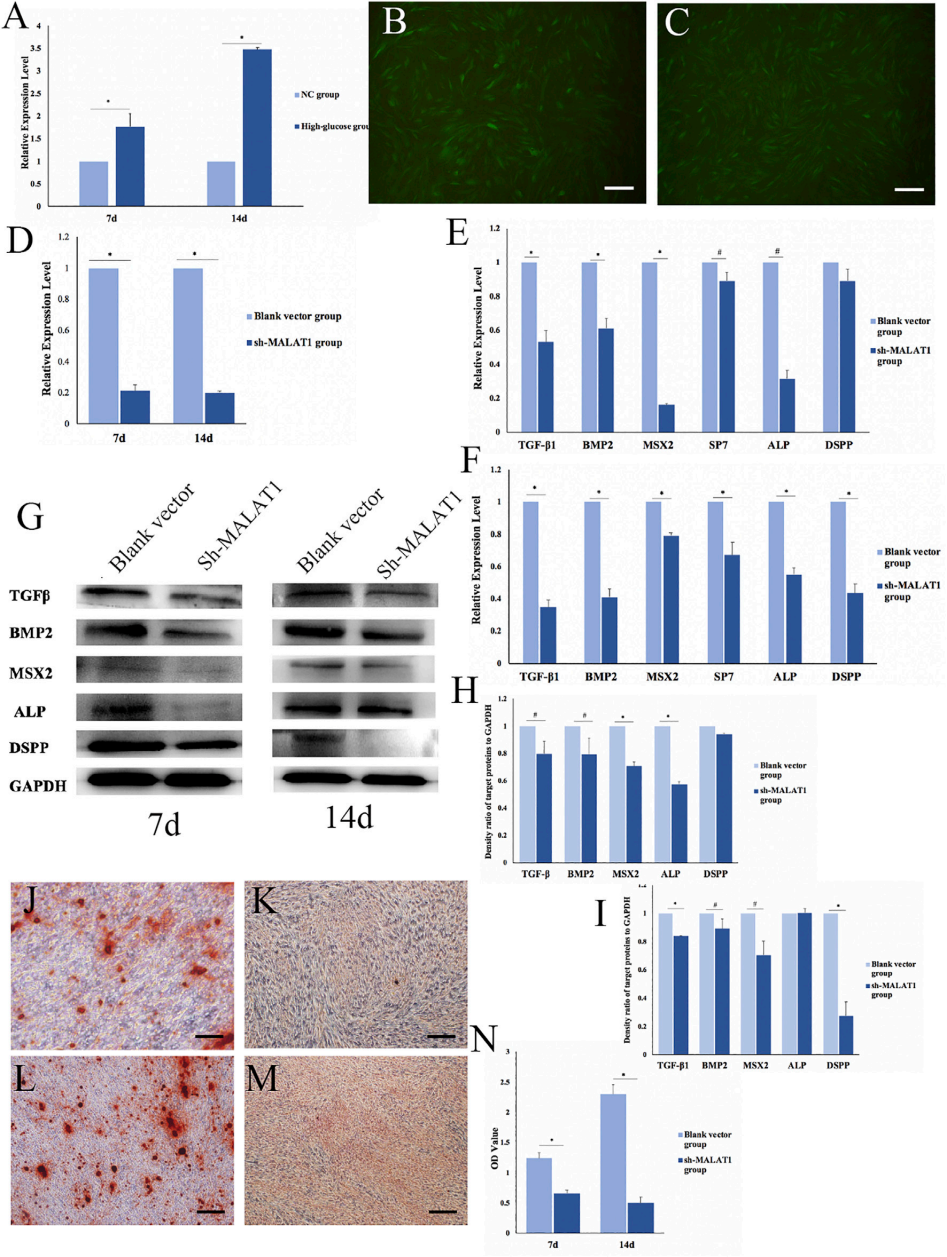
Effects of LncRNA-MALAT1 on mineralization of DPCs. **(A)** Expression of MALAT1 in DPCs which were cultured in high-glucose and induction of mineralization by qRT-PCR. **(B)** DPCs were cultured in DMEM, when confluence with 80% added blank vector and transfected for 72 h; **(C)** DPCs were cultured in DMEM, when confluence with 80% added sh-MALAT1 and transfected 72 h; **(D)** qRT-PCR detected the expression of MALAT1 after transfection with sh-MALAT1 and blank vector. **(E,F)** Expression of mineralization-related factors 7 days/14 days after transfection; **(G)** expression of mineralization-related factors detected by Western blot 7 days or 14 days after transfection; **(H,I)** density ratio of target proteins to GAPDH on days 7 and 14 . **(J,L)** Blank vector group 7 days/14 days after induced mineralization stained by alizarin red; **(K,M)** sh-MALAT1 group 7 days/14 days after induced mineralization stained by alizarin red; **(N)** semi-quantitative analysis of alizarin red staining (*n* = 3, scale bar = 500 μm ^*#*^ *p < 0.05,* *p < 0.01).

The expression of MALAT1 in DPCs after mineralization in an HG microenvironment was analyzed *via* qRT-PCR. The results showed significantly more MALAT1 expression in the HG group than in the NC group 7 and 14 days after mineralization induction (*p < 0.01; Figure 4A). Therefore, DPCs cultured in a high-glucose microenvironment overexpressed MALAT1 during mineralization induction.

We transfected DPCs with short hairpin (sh)-MALAT1 into DPCs, and the transfection efficiency reached 80% using P infection fluid at an MOI of 30, without affecting the cell status (Figures 4B,C). The transfection effects identified *via* qRT-PCR showed that MALAT1 expression was significantly decreased in DPCs transfected with sh-MALAT1 and the blank vector (*p < 0.01) (Figure 4D).

We transfected DPCs with sh-MALAT1 or the blank vector, and then analyzed the expression of mineralization-related factors using qRT-PCR. The results showed that only TGFβ-1, BMP2, MSX2, SP7, ALP, and DSPP were altered. The expression of mineralization-related factors was decreased in DPCs transfected with sh-MALAT1. MSX2 and ALP expression decreased the most on day 7 of mineralization, whereas TGFβ-1, BMP2, and DSPP were downregulated the most on day 14 (^*#*^ *p < 0.05,* *p < 0.01 vs. blank vector; Figures 4E,F). The results of Western blots of the expressed related factors were consistent with those of qRT-PCR (Figure 4G) and the results were also quantified using ImageJ software (^*#*^ *p < 0.05,* *p < 0.01; Figures 4H,I). Thus, LncRNA-MALAT1 promoted mineralization, whereas MALAT1 knockdown inhibited mineralization in DPCs. Alizarin red staining revealed no obvious mineralized nodule formation in DPCs upon MALAT1 knockdown (Figures 4J–M). Our semi-quantitative findings indicated a significant difference in the OD values between the HG and NC groups, which was consistent with the results of alizarin red staining (*p < 0.01; Figure 4N).

In a previous study, the involvement of MALAT1 in the mineralization of DPCs was examined; however, there is still much to be learned about the exact mechanism behind the regulation process.

## Discussion

LncRNAs are non-coding RNAs with a complex structure and diverse functions. They can be involved in protein recognition, catalysis, metabolism, embryonic stem cell differentiation, cell cycle regulation, tumorigenesis, and other physiological or pathological processes (Graf et al., 2013; Gu et al., 2019; Zhao L. et al., 2020; Qin et al., 2021). LncRNAs play important roles in many activities, such as dose compensation, epigenetic, cell cycle, and cell differentiation regulation. They participate in regulating gene expression at transcriptional and post-transcriptional levels, and through apparent modification. Because they are involved in these important regulatory processes, they are associated with almost all physiological or pathological processes, such as stem cell maintenance and differentiation, embryonic development, cell proliferation and apoptosis, cell metabolism, and immune regulation.

MALAT1 is an important LncRNA. Previous studies have shown that MALAT1 can specifically recruit serine/arginine-rich (SR) proteins and participate in epigenetic regulation and cell cycle regulation (Tripathi et al., 2013; Yang et al., 2013). The study revealed that MALAT1 is upregulated in lens epithelial cells (LECs) under high glucose conditions (Ye et al., 2020). High levels of Yap1 expressed in the retinas of mice with diabetic retinopathy upregulate MALAT1 and target VEGFA by regulating miR-200b-3p. When Yap1 is knocked down, the proliferation, migration, and angiogenesis of retinal microvascular endothelial cells in mice with diabetic retinopathy are reduced (Han et al., 2020). Many studies have found that an HG microenvironment can induce mineralization of dental pulp tissue and dental pulp cells as we have found in this study. BMP2 is upregulated in the vascular wall of patients with diabetes, leading to osteoblast differentiation and ectopic vascular calcification (Ilić et al., 2012). BMP2 belongs to the transforming growth factor *β* (TGF-β) family, which plays an important role in odontogenic differentiation. The expression of osteopontin (OPN) and ALP induced by 50 mmol/L glucose in rat dental pulp cells significantly increases, indicating that an HG microenvironment can increase the ability of DPCs to form hard tissue (Inagaki et al., 2010). High glucose levels can increase the expression of OPN, osteocalcin (OCN), and ALP in rat dental pulp cells (Takemoto et al., 2000; Giachelli et al., 2005; Hamamoto et al., 2010; Inagaki et al., 2010). Some studies have also pointed out that diabetic rats are more likely to have pulp stones and dentin thickening, and an increased area of OPN around the pathological calcified area of dental pulp tissues (Inagaki et al., 2010). The present findings are consistent with these. Osteogenic differentiation includes cell proliferation, extracellular matrix (ECM) maturation, and matrix mineralization (Zhao et al., 2020). The expression of genes encoding proteins involved in extracellular matrix maturation increased during extracellular matrix maturation, particularly that of ALP. However, ALP expression decreases at the late stage of osteoblast differentiation, and the expression of OPN, OCN, DSPP, and DMP1 gradually increases in the extracellular matrix (Li et al., 2011). Although, in our study, we found that an HG microenvironment promotes DPC mineralization and increases the expression of the mineralization-related factors in DPCs when we simulated an HG microenvironment *in vitro* using 50 mmol/L glucose, the specific regulatory mechanism remains unclear. Osteogenic differentiation is mainly regulated by tissue-specific transcription factors and epigenetic factors, and MALAT1 may exert a role in the regulation of osteogenic differentiation. However, whether MALAT1 is involved has not been established.

Next, our results found that high levels of LncRNA-MALAT1 were expressed in DPCs cultured in an HG microenvironment and participated in the process of HG regulation of DPC mineralization. The HG microenvironment in hippocampal cells and MALAT1 knockdown prevented HG-induced activation of mTOR and inhibited tau phosphorylation (Lu et al., 2021). Also, MALAT1 levels were increased in the renal tissues of diabetic rats and HG-treated cells (human renal tubular epithelial cells (HK-2 and 293T)), which were damaged by MALAT1 overexpression *via* the miR-2355-3p/IL6ST axis (Huang et al., 2021). In our study, the HG microenvironment increased the levels of MALAT1 in DPCs, was mainly expressed in the cytoplasm and may be involved in the processes of mineralization. Our results showed that inhibiting MALAT1 inhibited the mineralization of DPCs and regulated the expression of several mineralization-related factors (TGF-β1, BMP2, MSX2, SP7, ALP, and DSPP). In addition, MALAT1 can regulate cell mineralization and is associated with osteoporosis. Inhibiting the expression of MALAT1 can reduce ALP activity and inhibit osteogenic differentiation of bone marrow mesenchymal stem cells (BMSCs) by enhancing the activation of the MAPK signaling pathway that promotes the progression of osteoporosis (Zheng et al., 2019). The results showed significantly increased MALAT1 expression in DPCs. Mineralization was inhibited in DPCs when transfected with sh-MALAT1. Our experimental results are consistent with those reported in the aforementioned studies.

Then, how does MALAT1 regulate the osteogenic differentiation of DPCs? In our study, we find that MALAT1 is expressed in the cytoplasm of DPCs. It is crucial to know the location of LncRNA to understand how it functions and what its regulatory mechanism is. It is likely that MALAT1 was involved in biological functions in the cytoplasm rather than transcription regulation, such as acting as a ceRNA. Salmena et al. (2011) presented the idea of competitive endogenous RNA (ceRNA) in 2011. Their hypothesis proposed a network comprising lncRNAs, circular RNAs (circRNAs), and mRNAs, and that lncRNAs could regulate the role of mRNAs by competing for one or more miRNAs. Many studies show that MALAT1 can function as a ceRNA and interacts with miRNAs. The expression of MALAT1 and RUNX2 was significantly increased, while the expression of miR-30 was downregulated in adipose-derived mesenchymal stem cells induced in an osteogenic differentiation medium. LncRNA-MALAT1 promoted the RUNX-mediated osteogenic differentiation of adipose-derived mesenchymal stem cells by targeting miR-30 (Yi et al., 2019). The expression of MALAT1 was increased while that of miR-143 was decreased after osteogenic induction of hBMSCs. miR-143 could inhibit OSX and affect the expression of ALP, OCN, and OPN. These results indicated that MALAT1 regulates the expression of OSX by targeting miR-143 (Gao et al., 2018). MALAT1 acts as a sponge molecule of miR-204 and upregulates the expression of SMAD4 and the activation of SMAD4 promotes the expression of ALP and OCN, thus promoting bone formation and mineralization (Xiao et al., 2017). *In situ* hybridization showed that MALAT1 was mainly expressed in the cytoplasm of DPCs in our study. MALAT1 may participate in DPC mineralization through miRNA interaction, but the specific regulation processes and pathways warrant follow-up studies. Thus, we believe that MALAT1 is the target of regulating odontogenic differentiation, and we will explore its regulatory mechanism in the following experiments. Nevertheless, we are planning to conduct related *in vivo* experiments to investigate the molecule mechanism in the future.

## Conclusion

In conclusion, we found that an HG microenvironment maintains the colony-forming ability of DPCs but inhibits their proliferative capacity. After mineralization induction, an HG microenvironment can upregulate the expression of LncRNA-MALAT1 in DPCs, and MALAT1 may target mineralization-related factors (TGF-β1/BMP2-MSX2-ALP/DSPP) to promote DPC mineralization.

## Data Availability

The original contributions presented in the study are included in the article/Supplementary Material; further inquiries can be directed to the corresponding author.
