# Supplementary figures and images for: Effect of LncRNA-MALAT1 on mineralization of dental pulp cells in a high-glucose microenvironment
Source: Front Cell Dev Biol. 2022 Aug 11;10:921364. doi: 10.3389/fcell.2022.921364 (PMC9402893; doi:10.3389/fcell.2022.921364)

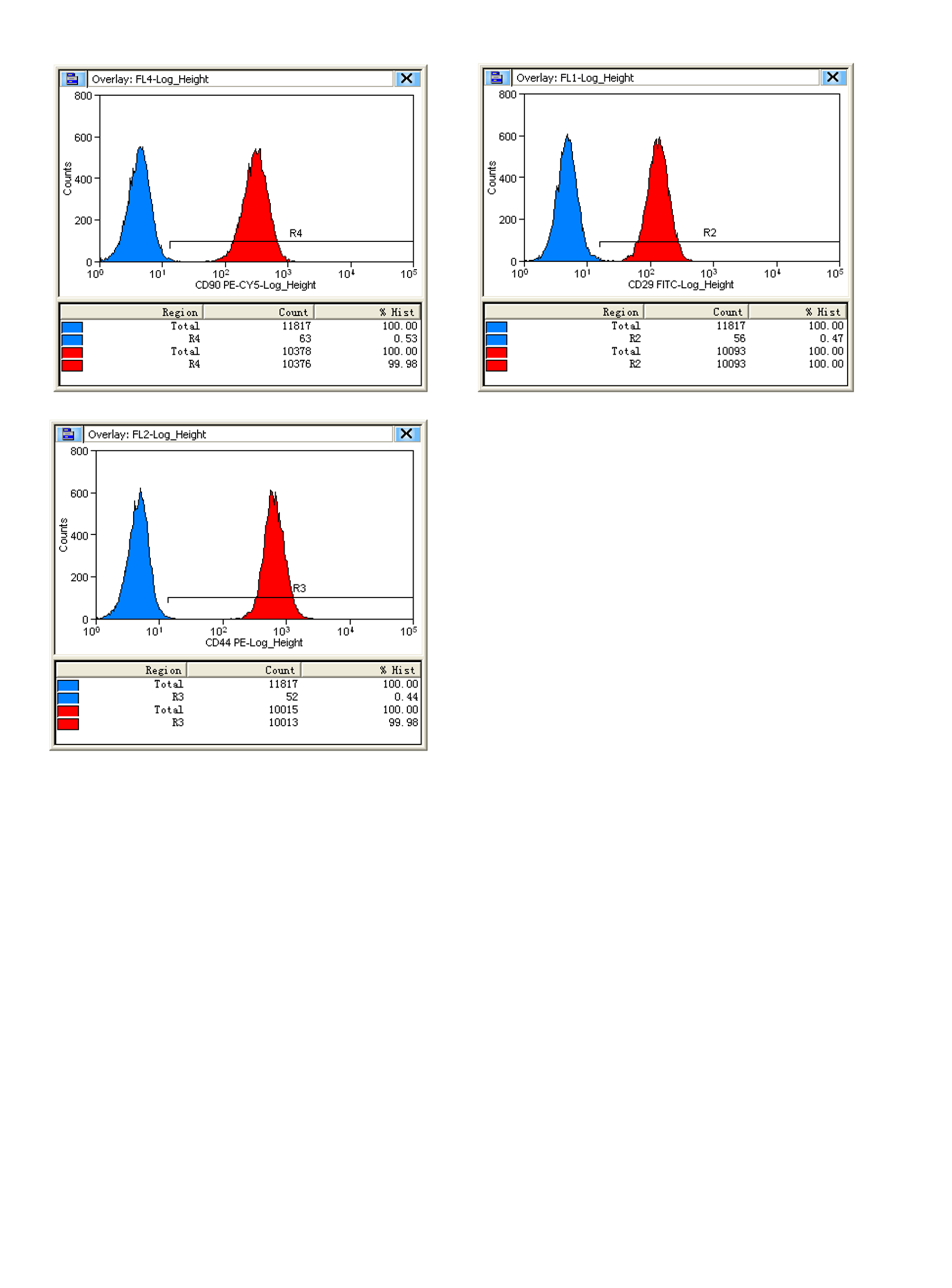

Supplement: Supplementary file 1 [file Image1.TIF]
